# Protective Effects and Target Network Analysis of Ginsenoside Rg1 in Cerebral Ischemia and Reperfusion Injury: A Comprehensive Overview of Experimental Studies

**DOI:** 10.3390/cells7120270

**Published:** 2018-12-12

**Authors:** Weijie Xie, Ping Zhou, Yifan Sun, Xiangbao Meng, Ziru Dai, Guibo Sun, Xiaobo Sun

**Affiliations:** 1 Beijing Key Laboratory of Innovative Drug Discovery of Traditional Chinese Medicine (Natural Medicine) and Translational Medicine, Institute of Medicinal Plant Development, Peking Union Medical College and Chinese Academy of Medical Sciences, Beijing 100193, China; ginseng@163.com (W.X.); zhoup0520@163.com (P.Z.); xbmeng@implad.ac.cn (X.M.); athenadai219@163.com (Z. D.); 2 Key Laboratory of Bioactive Substances and Resource Utilization of Chinese Herbal Medicine, Ministry of Education, Beijing 100193, China; 3 Key Laboratory of Efficacy Evaluation of Chinese Medicine against Glycolipid Metabolic Disorders, State Administration of Traditional Chinese Medicine, Beijing 100193, China; 4 Zhongguancun Open Laboratory of the Research and Development of Natural Medicine and Health Products, Beijing 100193, China; 5 Institute of Medical Information, Chinese Academy of Medical Sciences, Beijing 100020, China; sun.yifan@imicams.ac.cn

**Keywords:** ginsenoside Rg1, ischemia stroke, cerebral ischemia and reperfusion injury, anti-inflammatory, anti-oxidant, proliferation, differentiation, energy metabolism, review

## Abstract

Cerebral ischemia-reperfusion is a complicated pathological process. The injury and cascade reactions caused by cerebral ischemia and reperfusion are characterized by high mortality, high recurrence, and high disability. However, only a limited number of antithrombotic drugs, such as recombinant tissue plasminogen activator (r-TPA), aspirin, and heparin, are currently available for ischemic stroke, and its safety concerns is inevitable which associated with reperfusion injury and hemorrhage. Therefore, it is necessary to further explore and examine some potential neuroprotective agents with treatment for cerebral ischemia and reperfusion injury to reduce safety concerns caused by antithrombotic drugs in ischemic stroke. Ginseng Rg1 (G-Rg1) is a saponin composed of natural active ingredients and derived from the roots or stems of *Panax notoginseng* and ginseng in traditional Chinese medicine. Its pharmacological effects exert remarkable neurotrophic and neuroprotective effects in the central nervous system. To explore and summarize the protective effects and mechanisms of ginsenoside Rg1 against cerebral ischemia and reperfusion injury, we conducted this review, in which we searched the PubMed database to obtain and organize studies concerning the pharmacological effects and mechanisms of ginsenoside Rg1 against cerebral ischemia and reperfusion injury. This study provides a valuable reference and clues for the development of new agents to combat ischemic stroke. Our summarized review and analysis show that the pharmacological effects of and mechanisms underlying ginsenoside Rg1 activity against cerebral ischemia and reperfusion injury mainly involve 4 sets of mechanisms: anti-oxidant activity and associated apoptosis via the Akt, Nrf2/HO-1, PPARγ/HO-1, extracellular regulated protein kinases (ERK), p38, and c-Jun N-terminal kinase (JNK) pathways (or mitochondrial apoptosis pathway) and the caspase-3/ROCK1/MLC pathway; anti-inflammatory and immune stimulatory-related activities that involve apoptosis or necrosis via MAPK pathways (the JNK1/2 + ERK1/2 and PPARγ/HO-1 pathways), endoplasmic reticulum stress (ERS), high mobility group protein1 (HMGB1)-induced TLR2/4/9 and receptor for advanced glycation end products (RAGE) pathways, and the activation of NF-κB; neurological cell cycle, proliferation, differentiation, and regeneration via the MAPK pathways (JNK1/2 + ERK1/2, PI3K-Akt/mTOR, PKB/Akt and HIF-1α/VEGF pathways); and energy metabolism and the regulation of cellular ATP levels, the blood-brain barrier and other effects via N-methyl-D-aspartic acid (NMDA) receptors, ERS, and AMP/AMPK-GLUT pathways. Collectively, these mechanisms result in significant neuroprotective effects against cerebral ischemic injury. These findings will be valuable in that they should further promote the development of candidate drugs and provide more information to support the application of previous findings in stroke clinical trials.

## 1. Introduction

Stroke is one of the leading causes of death worldwide. Nearly 6 million people die from stroke each year, and it is estimated that the lifetime risk for stroke is 8% to 10%. Ischemic stroke accounts for 85% of all strokes, while hemorrhagic stroke accounts for 15% [1]. The hazards associated with ischemic stroke are mainly caused by cerebral ischemia and reperfusion injury (CI/RI), which is a pathological condition characterized by an initial restriction of blood supply to an organ followed by the subsequent restoration of perfusion and concomitant reoxygenation [2,3]. Additionally, ischemia and reperfusion injury contribute to pathologies under a wide range of conditions, mainly including energy metabolism disorders, oxidative stress, inflammatory stress [4,5] and cytokine damage glutamate toxicity, Ca^2+^ overload, excessive nitric oxide (NO) synthesis, apoptosis, and many other factors [3,4,5,6,7,8]. CI/RI and the secondary damage it causes to brain tissues are closely associated with immunity and inflammation responses [4,5]. In past decades, to explore better treatment options for ischemic stroke and reperfusion injury, researchers have carried out extensive and in-depth studies. More than 1000 drugs have been tested, with over 400 demonstrating efficacy in animal models of stroke; furthermore, substantial efforts have been made to explore preventive methods to reduce the morbidity and mortality of stroke [7], resulting in the development of recombinant tissue plasminogen activator (r-TPA), aspirin and heparin [3,5]. However, most of these treatments have disappointingly been found to be ineffective during the acute phase of stroke, and intravenous recombinant tissue plasminogen activator (r-tPA) is currently the only approved agent for the treatment of acute ischemia stroke [9], and it has safety concerns associated with reperfusion injury and hemorrhage. Therefore, it is necessary to examine some potential neuroprotective agents for their ability to treat ischemic stroke.

*Panax notoginseng* (Burk) F. H. Chen and *Panax ginseng* C. A. Mey are two commonly used Chinese medicinal herbs, the roots or stems of which have been used for the treatment of cardiovascular disease in many Asian countries for several hundred years [10]. Pharmacological studies have shown that *P. notoginseng*, *P. ginseng*, and their extracts, panax notoginseng saponins (PNS) and ginseng total saponins (GTS), exert multiple pharmacological activities, such as anti-inflammatory [11], anti-oxidative [12,13], platelet aggregation-inhibiting, and neuronal apoptosis-suppressing effects [14].

**Figure 1:**
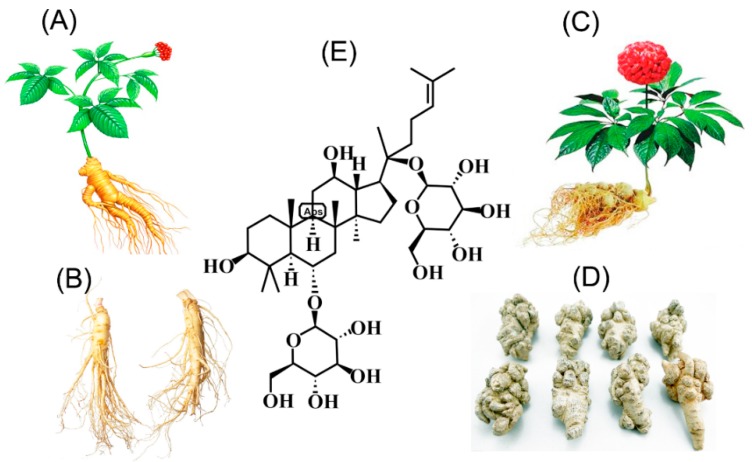
Sources and chemical structure of ginsenoside Rg1 obtained from *Panax notoginseng* and *P. ginseng*. (**A**) stems and leaves of *P. ginseng*; (**B**) roots of *P. ginseng*; (**C**) stems and leaves of *P. notoginseng*; (**D**) roots of *P. notoginseng*; (**E**) chemical structure of ginsenoside Rg1.

As shown in Figure 1, ginsenoside Rg1 (G-Rg1) is a tetracyclic triterpenoid mainly derived from the roots or stems of *P. notoginseng* and *P. ginseng* that is obtained via an extraction and purification processes (Figure 1) and chemically belongs to the PPT ginsenoside group [15]. The G-Rg1 content was determined via simple and accurate HPLC or UPLC methods and found to account for 0.22% ± 0.02% of sun-cured *ginseng*, 0.64% ± 0.004% of the stems and leaves of *ginseng* [16,17,18], 4.41% ± 0.05% of the roots or stems of *P. notoginseng*, and 3.21% ± 0.08% of the roots or stems of *P. notoginseng* [19]. These data indicate that the G-Rg1 content is clearly higher in *P. notoginseng* than in *P. ginseng* and that the stems and leaves seem to have more value. Additionally, G-Rg1 is regarded as one of the main bioactive compounds responsible for the pharmaceutical actions of ginseng, which show little toxicity, and some evidence has shown that its pharmacological effects are remarkable in that they include neurotrophic and neuroprotective effects on the central nervous system [20,21,22,23,24,25,26,27,28,29]. Most importantly, as a tetracyclic triterpenoid (Figure 1E) found in natural medicinal plants, G-Rg1 could promote hippocampal neurogenesis, improve neuroplasticity, enhance learning and memory, exert anti-aging [30] and antifatigue effects, and regulate immunity and antitumor activity [31,32,33,34,35]. Additionally, an increasing amount of evidence indicates that G-Rg1 exerts neuroprotective effects both in vivo and in vitro [30,31,32,33,34,35,36,37]. Various mechanisms have been shown to underlie G-Rg1 activity [38,39], including the activation of anti-oxidant, immune stimulatory, anti-inflammatory and anti-apoptotic activities, effects on nerve growth factors, the inhibition of excitotoxicity, the induction of excessive Ca^2+^ influx into neurons, the preservation of the structural integrity of neurons, and the maintenance of cellular adenosine triphosphate (ATP) levels.

However, to date, no systematic review has been conducted to assess the protective effects of and mechanisms underlying how G-Rg1 combats cerebral ischemia/reperfusion injury (CI/RI). A systematic review of all evidence available from animal experiments preceding clinical trials would provide an adequate interpretation of the limitations and potential of novel treatment strategies. Moreover, while various candidate drugs have failed to treat cerebral ischemia, those studies have prompted series of suggestions that could improve the likelihood of successful translation. Among these is that if a systematic review and analysis of preclinical studies of alternative active ingredients was to be carried out, it would likely promote candidate drug development and provide more information from the previous literature that could be used as a bridge into clinical trials of stroke. Therefore, in the present study, we conducted a systematic review of all available animal studies to evaluate the preclinical evidence related to G-Rg1 in experimental CI/RI studies.

To explore and summarize the protective effects and relevant mechanisms of ginsenoside Rg1 against CI/RI, we conducted this review by searching the “PubMed” database via using “Ginsenoside Rg1” and “Ischemia” as search terms to obtain the literature concerning animal experiments in latest 10 years (https://www.ncbi.nlm.nih.gov/pubmed/?term = ((ginsenoside ± Rg1%5BTitle%2FAbstract%5D) ± AND ± ischemia)). This allowed us to organize and analyze the literature concerning the pharmacological effects and mechanisms of ginsenoside Rg1 against CI/RI, which will be valuable for further promoting candidate drug development and providing more citation-based information that can be applied in clinical trials of stroke.

## 2. Protective Effects and Mechanisms

### 2.1. Regulation of Oxidative Stress and Apoptosis

Compared with other organs in the human body, brain tissues and neurons are more oxygen-consuming and more prone to produce higher levels of reactive oxygen species (ROS), reactive nitrogen species (RNS), and unsaturated fatty acids than can be rapidly oxidized, whereas the anti-free radical system of nervous tissues are relatively weaker, indicating that neurons are more sensitive to oxidative stress than is observed in other tissues. Active radicals include superoxide anion (O^2−^), hydrogen peroxide (H_2_O_2_), nitric oxide (NO), and hydroxyl radicals (OH-) [40,41]. Ischemia and reperfusion (I/R) injury has been closely related to increased ROS and platelet levels and leukocyte activation and altered calcium homeostasis [34,42,43,44,45,46]. After ischemia-reperfusion, the production of ROS increases, leading to the damage of intracellular biofilm lipids (MDA), proteins and nucleic acids, mitochondrial damage, and the evoked release of apoptosis inducer (AIF) and cytochrome C (Cyt-C) in mitochondria. Additionally, these increasing levels of intracellular AIF and Cyt-C induce apoptosis via cell signaling pathways, resulting in the activation of a downstream apoptotic cascade that includes the activation of cleaved caspase-3 (CC-3) and cleaved caspase-9 (CC-9), both of which regulate the levels of anti-apoptotic proteins [47,48,49,50,51]. Additionally, during this process, ROS cause damage to biofilm substances, resulting in increases in MDA and lactate dehydrogenase (LDH) leakage, which are important indicators of the severity of neuronal cell injury by free radicals. Currently, various animal models of cerebral ischemia have been applied, and ginsenoside Rg1 (G-Rg1) has been demonstrated to inhibit CI/RI by regulating oxidative stress, as shown in Table 1.

First, various animal models of cerebral ischemia have been tested, with results demonstrating that G-Rg1 may exert its neuroprotective activities on CI/RI by regulating oxidative stress [15,38,52,53,54,55], as shown in cerebral I/R (CI/R)-induced C57BL/6 mouse models [55], middle cerebral artery occlusion (MCAO/R)-induced SD rat models [56], and transient middle cerebral artery occlusion (t-MCAO)-induced BALB/c mouse models [24,56]. As shown in Table 1, Rg1 treatment greatly improved neurological function, significantly diminished brain edema, neurological deficit scores, and infarct volume in the model mice or rats, clearly inhibited LDH leakage, MDA, and nitric oxide (NO), and prominently increased the activities of superoxide dismutase (SOD), MPO, GSH-Px, and CAT enzymes, thereby stabilizing mitochondrial membrane potential [57,58] and reducing neuronal apoptosis [36,59]. Concentrations of G-Rg1 of approximately 0.1–10 mM have been shown to remarkably reduce cytotoxicity-induced oxidative stress [59].

Second, as shown in Table 1, the pre-protective and neuroprotective effects of G-Rg1 administration and treatment in ischemic injury have been verified in various oxidative stress-induced cell models, such as oxygen glucose deprivation (OGD)-induced cortical neuron models [24], OGD-induced neural stem cell (NSC) models [36], H_2_O_2_-induced SH-SY5Y cell injured models [60], H_2_O_2_-treated PC12 cell models, and human vascular endothelial EA.hy926 cells [61]. Compared to results obtained in control groups, all of these experimental models have indicated that G-Rg1 significantly reduced OGD or H_2_O_2_-induced cell apoptosis in cortical pyramidal cells, NSCs, SH-SY5Y, or PC12 cells; markedly elevated cell viability and survival rates in various cells (NSCs, SH-SY5Y, PC12 cells, or EA.hy926 cells) treated with OGD or H_2_O_2_; diminished the amount of ROS production and the levels of MDA, NO, leaked LDH and intracellular Ca^2+^ [62,63]; increased the activities of SOD, GSH-Px and CAT enzyme; reduced the expression of pro-apoptotic proteins cleaved caspase-3 (CC-3) and Bax; and increased the expression of the anti-apoptotic protein Bcl-2 [24,60,61,63]. Additionally, H_2_O_2_-induced injuries in mouse cultured astrocytes were prevented by G-Rg1 because it inhibited ROS production, intracellular Ca^2+^ overload, and the loss of mitochondrial membrane potential (MMP) [62,64], indicating that G-Rg1 may reduce mitochondrial damage and suppress the mitochondrial apoptosis pathway.

In summary, the main mechanisms by which G-Rg1 exerts its significant neuroprotective effects in cerebral ischemic injury are closely associated with anti-oxidation and the inhibition of apoptosis. Previously published studies have confirmed that G-Rg1 attenuates OGD-induced oxidative stress and regulates the Nrf2/HO-1 pathways in modeled animals and cells [24,56] and modulates the expression levels of PPARγ/HO-1, indicating that G-Rg1 downregulates Nrf2 levels in the cytoplasm, upregulates Nrf2 levels in the nucleus, and elevates the mRNA and protein levels and the rate of nuclear translocation of HO-1 [62]. Additionally, treatment with G-Rg1 may have altered the levels of bcl-2, CC-3, and cleaved caspase-9 (CC-9) in modeled animals and cells [24,56], reduced the expression levels of the pro-apoptotic proteins cleaved CC-3 and Bax, elevated the expression of the anti-apoptotic protein Bcl-2, suppressed caspase-3 immunoreactivity, and contributed to heat shock protein 70 (HSP70) gene expression in a dose-dependent manner [60]. Moreover, G-Rg1 may reduce I/IR-induced oxidative stress by inhibiting the expression of p-p38 and p-JNK2 and regulating p38/JNK2 phosphorylation in H_2_O_2_-induced PC12 cells [61], OGD-treated NSCs [36], and an H_2_O_2_-induced SH-SY5Y model of cell injury [60]. Hence, these studies provide solid evidence for the neuroprotective effects of G-Rg1 and reveal the mechanisms underlying anti-oxidation and the inhibition of apoptosis via the Akt, Nrf2/HO-1, PPARγ/HO-1, ERK, p38, and JNK MAPK signaling pathways or mitochondrial apoptosis pathway and the caspase-3/ROCK1/MLC pathway [65].

### 2.2. Regulation of Necrosis and Apoptosis Associated with Anti-Inflammatory Activity

Immunity and inflammation are key elements that contribute to the pathobiology of stroke, and CI/RI and the secondary damage it causes to brain tissues are closely associated with immunity and inflammation responses. Increasing evidence has indicated that an inflammatory response is involved in all stages of ischemia-reperfusion injury [68,69]. Once ischemia/reperfusion occurs, ROS production promotes the activation of complements, platelets and endothelial cells; activates inflammatory transcription factors and the release inflammatory signals [70]; and generates inflammatory factors, including IL-6, IL-1β, and TNF-α [68,69,70,71]. At the same time, because ROS causes neuronal cell death and the release of nucleosides, they can activate purine receptors on microglia and macrophages, leading to the aggregation of and infiltration by inflammatory cells to simultaneously induce inflammation and a series of secondary tissue damage, such as the destruction of the blood-brain barrier and cerebral edema [15,43,72]. Nuclear factor-κB (NF-κB) plays an important role during this process. When NF-κB binds to the target site in the nucleus, it initiates the transcription and regulates the expression of cytokines and therefore inflammatory reactions. At the same time, inflammatory factors and adhesion molecules can induce the further activation of NF-κB and aggravate the inflammatory process [68,69,72].

Coincidentally, multiple in vitro and in vivo experiments have shown that G-Rg1 may reduce ischemia-reperfusion injury by inhibiting necrosis and apoptosis associated with anti-inflammatory activity [24,35,36,73,74,75,76,77,78]. First, in vivo ischemia-reperfusion injury models carried out using a cerebral I/R-induced C57BL/6 mouse model [26], an MCAO-induced male rat model [79] and a middle cerebral artery I/R injury rat model [24,26] demonstrated that between MCAO rats treated with or without G-Rg1, the G-Rg1 group (40 mg/kg, oral administration; 30 mg/kg, tail vein injection), the infarct volume, brain edema, neurological deficit scores, and neurological function were significantly improved; the serum levels of released NO, interleukin-1β (IL-1β) [56], tumor necrosis factor alpha (TNF-α) and interleukin-6 (IL-6) were lower [26]; the levels of TNF-α and ICAM-1 mRNA were lower; the content and expression of TGF-β1 and brain-derived neurotrophic factor (BDNF) were higher in the CA1 region of the hippocampus; the neurocyte survival rate was higher [26]; the rate of apoptosis was lower; the level of caspase-3 protein was lower [80], and the number of CD11b-positive cells and miR-155-5p levels were lower [80]. These findings indicate that G-Rg1 acts to inhibit inflammation and associated apoptosis in in vivo models of cerebral IR.

Moreover, in in vitro experiments, treatment with G-Rg1 was demonstrated to significantly relieve the inflammatory response that occurred after hypoxic injury by reducing multiple indicators of inflammation in an OGD-induced cortical neurons model [24]; by increasing cell viability, reducing NO levels and the content and expression of TNF-α while upregulating the content and expression of TGF-β1 in BV2 microglial cells in an OGD-injured microglia model [66,81]; by changing the expression levels of PPARγ, bcl-2, bax, CC-3, CC-9, IL-1β, and HMGB1 and suppressing the expression levels of miR-155-5p, pri-miR-155, and pre-miR-155 in BV2 microglial cells injured by OGDs [66,79]; and by inhibiting the phosphorylation of NF-κB, p50, p65, and IKKα/β in EA.hy926 cells treated with TNF-α, as shown in Table 2. Additionally, G-Rg1 ameliorates BBB permeability, reduces the risk of cerebral edema and cerebral hemorrhage, and downregulates the expression of PAR-1 [38], which is closely related to other adhesion molecules, such as ICAM-1, MMP-9 and MMP-2.

The studies evaluated here (Table 2) demonstrate that treatment with G-Rg1 significantly reduced the expression of TNF-α, IL-1β, and IL-6 in ischemia-reperfusion animal and cell models in addition to the number of IL-1β-positive cells. Additionally, treatment with G-Rg1 reduced the levels of microRNA (miR)-155-5p and CD11b in OGD-induced BV2 cells [79]; activated PPARγ signaling, which was inhibited by GW9662 (a selective PPARγ antagonist) [24]; inhibited the phosphorylation of NF-κB, p50, p65, and IKKα/β induced by treatment with TNF-α [61]; and strengthened protection against cerebral ischemia injury via anti-apoptotic and anti-inflammatory effects and reduced the phosphorylation of JNK1/2, HMGB1, and RAGE in the hippocampus of model animals. These mechanisms might be associated with an ability to restrict the activation of the NF-κB [59] and JAK1/STAT1 signal pathways, the ability to regulate endoplasmic reticulum stress (ERS) after cerebral ischemia [80], and the ability to regulate the kinase 1/2 (ERK1/2) [59], PPARγ/HO-1 pathways [24], and Akt pathways [59].

### 2.3. Regulation of the Neural Cycle, Proliferation, Differentiation, and Regeneration

It is well known that the MAPK/ERK pathway is involved in cell proliferation, differentiation, senescence, and apoptosis, and MAPK/p-38 is activated by various inflammatory extracellular mediators, whereas JNK isoforms are strongly activated during various cellular stress responses. These findings indicate that in cortical neurons, the phosphorylated (activated) status of p38 and ERK-1/2 is upregulated in the absence of oxygen, whereas in rat hippocampal slices, MAPK (p38) and MAPK (ERK1/2) and the phosphorylation status of ERK-1 are upregulated in response to hypoxia, but existing tests found this upward trend of phosphorylation was downregulated by G-Rg1 [82,83,84,85,86,87]. Additionally, Akt is a serine/threonine kinase also known as protein kinase B (PKB/Akt) that has been shown to act as a key regulator of cell survival, growth, apoptosis, and proliferation in the presence of growth factors and extracellular stimuli, especially in cerebral ischemia and reperfusion injury. And the activation of Akt is usually mediated by PI3K, which is recruited to the plasma membrane, where it binds to PI3K partners involved in the activation of phosphorylation sites, which also could be regulated [59,88,89].

In addition to its roles in the regulation of oxidative stress and inflammation, G-Rg1 also could regulate cytokine expression, the cell cycle, cell proliferation, cell differentiation, and cell apoptosis after stroke by activating PI3K-Akt/mTOR, mitogen-activated protein kinases (MAPKs) and ERS regulatory signaling pathways, as confirmed by the results of various types of testing models, such as the modified Rice–Vannucci model [90], hypoxia ischemia brain damage (HIBD)-induced SD rat models [91,92,93], an I/R-induced SD rat model [86], a MCAO followed by 24-h reperfusion (MCAO/R)-induced SD rat model [94], and a normal adult mouse and global ischemia gerbil model [30,95]. Additionally, the MAPK pathway is critical for the anti-cerebral ischemia effects of G-Rg-1 and is associated with the regulation of the NF-κB and HIF proteins in the studies evaluated here.

In HIBD-induced animals, G-Rg1 treatment enhanced the neural survival rate, reduced neurological impairment and pathologic damage [90,91], clearly decreased cell apoptosis and improved ischemic conditions, increased neural viability, promoted angiogenesis, induced neurogenesis [92], facilitated angiogenesis after HIBD in neonatal rats [93], increased the efficacy of and structures associated with neural plasticity, promoted the differentiation of transplanted BMSCs into neurons and glial cells [54], and increased the proliferation and differentiation of neural progenitor cells in the dentate gyrus of the hippocampus in normal adult mice and a gerbil model of global ischemia model [30]. These effects improved cerebral ischemia and recovery and reduced cell apoptosis (Table 3). Moreover, G-Rg1 increased the expression of BDNF and Bcl-2, enhanced the formation of new synapses, inhibited apoptosis and calcium overload and facilitated angiogenesis after HIBD and activities that have been tightly associated with the ability of G-Rg1 to regulate the expression levels of VEGF and Caspase-3 [91], inhibited the activation of Caspase-3 by the ERK1/2 signaling pathway [81], downregulated the expression of p-JNK in the hippocampal CA1 region, upregulated HIF-1α expression [54,91], and strengthened and stabilized the HIF-1alpha/VEGF signaling pathways after HIBD in neonatal rats [92,93]. Therefore, the pharmacological effects of Rg1 may be attributed to its ability to regulate the expression levels of HIF-1α, VEGF, BDNF, Caspase-3, PI3K-Akt/mTOR, PKB/Akt, JNK1/2, ERK1/2, JNK, and HIF-1α/VEGF. These signaling pathways are involved in increasing proliferation and differentiation in neural progenitor cells and play anti-apoptotic roles in HIBD, as shown in Table 3.

### 2.4. Regulation of Energy Metabolism and the Blood-Brain Barrier and Other Effects

In general, abundant blood must be supplied to the brain, with the cerebral blood flow accounting for 20% of cardiac blood output at rest. In cerebral ischemia, the blood flow to the brain is reduced or blocked, and the ischemic brain tissue area cannot obtain sufficient blood oxygen. Additionally, brain nerve tissue can only obtain energy through anaerobic sugar glycolysis, which reduces ATP production, increases adenosine diphosphate (ADP) production, and increases the ADP/ATP ratio, resulting in a lack of nutrients required for brain tissue metabolism, damage to mitochondria and other organelles, and further aggravation of brain tissue damage in ischemic areas [4]. Furthermore, after CI/RI, ATP production is insufficient and Na-K-ATPase activity is decreased, resulting in increased intracellular Na^+^ concentrations and consequential cytotoxic cerebral edema and nerve damage [5]. After G-Rg1 treatment, the levels of ATP and adenosine monophosphate (AMP) markedly increased, the levels of total adenine nucleotides (TANs) and energy charge (EC) improved [96], and mitochondrial transmembrane potential (MMP) increased [97]. The mechanisms underlying these effects might be associated with improving mitochondrial ultrastructure and oxidative respiratory function, which lead to the inhibition of mitochondrial apoptosis, enhance the expression of glucose transporter 3 (GLUT3), promote the activation of AMPKα1/2, increase the uptake of glucose into nerve cells, and increase the supply and intake of glucose, as shown in Table 4.

Moreover, glutamate (Glu) is one of the most widely distributed excitatory amino acids (EAAs) in the central nervous system. Under ischemia and hypoxia conditions, Glu is released in large quantities, resulting in the activation of Glu receptors, and causes irreversible damage or even death of nerve cells via two major mechanisms, including N-methyl-D-aspartate (NMDA) receptor-mediated delayed injury of nerve cells and non-NMDA receptor-mediated neuronal injury in the acute phase of ischemia [4,7,8]; these effects lead to decreased membrane permeability, Ca^2+^ influx, calcium overload, ROS aggregation, mitochondrial injury, brain barrier permeability, and cytotoxic brain edema, all of which result in cell necrosis and apoptosis. Nevertheless, G-Rg1 treatment restored the intracellular calcium overload in neurons, reduced the excitatory Glu toxicity induced by CI/RI by reducing the contents of Glu and Asp in serum [26], decreased the intracellular concentration of free calcium and nNOS activity after OGD exposure [98], enhanced iNOS activity in both the hippocampus and cortex [99], and blocked the overinflux of calcium into neuronal cells [98]. These potential mechanisms may be involved in regulating ERS and mediating iNOS activity and NMDA receptors, as shown in Table 4.

## 3. Conclusions and Remarks

Cerebral ischemia-reperfusion is a complicated pathological process. The damage and cascade of reactions caused by ischemia and reperfusion in brain tissues are related to decreased blood flow, ischemic-induced energy metabolism disorder, oxidative stress, inflammatory stress, cytokine damage, excitatory toxicity by glutamate, intracellular calcium overload, NO synthesis, and many other factors [2,3,4,5,6,7,100], even including some genetic disease as a possible complication, such as Fabry disease [100,101]. Moreover, the numerous abovementioned factors and mechanisms that lead to CI/RI are related to each other and can interact with and cause each other, eventually leading to apoptosis or nerve necrosis in the ischemic region [102,103].

**Figure 2:**
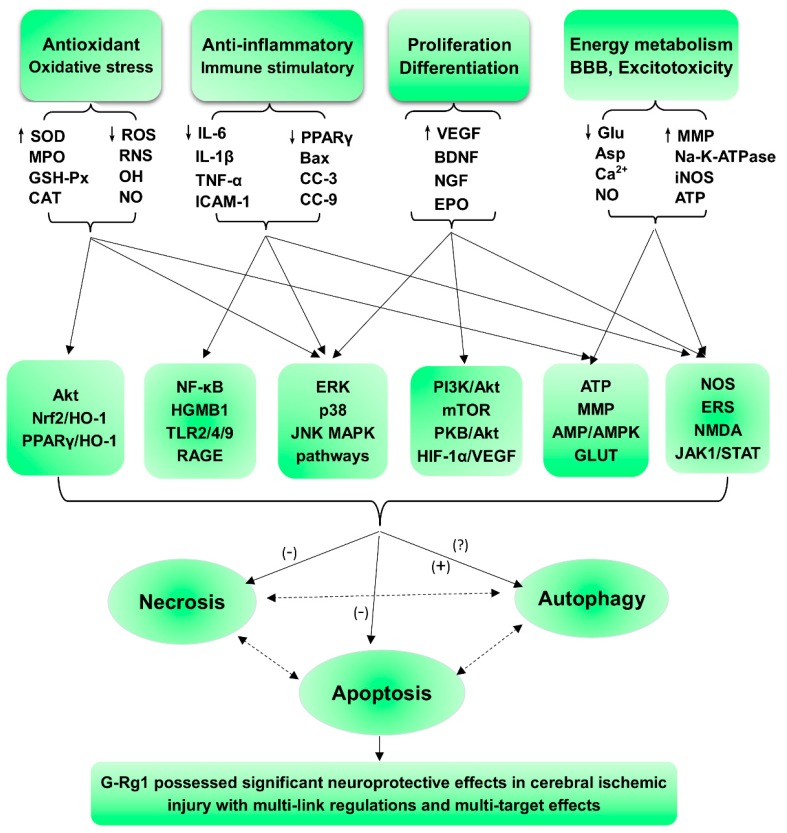
Summary and functional network target analysis of the ginsenoside Rg1, which possesses significant neuroprotective effects in cerebral ischemic injury and exhibits multiple links across regulatory mechanisms and multitarget effects. (−), downregulation or inhibition; (+), upregulation or activation; (?), uncertainty or undetermined.

Ginseng Rg1, a saponin obtained as a natural active ingredient in traditional Chinese medicine (TMR), is a traditional stem extract of ginseng and *Panax notoginseng*, and its pharmacological effects are remarkable in that it exerts neurotrophic and neuroprotective effects on the central nervous system. In our review, we summarize the protective effects of G-Rg1 against CI/RI in addition to the mechanisms underlying these effects. The results of our analysis show that 4 main mechanisms are involved (Figure 2): anti-oxidant and associated apoptotic effects; anti-inflammatory and immunostimulatory-related effects on apoptosis or necrosis; neurological cell cycle, proliferation, differentiation, and regeneration; and energy metabolism and regulation of cellular ATP levels, blood-brain barrier (BBB) permeability, excitatory amino acids (EAAs), and other processes, including the activation of nerve growth factor (NGF), excitotoxicity, and excessive Ca^2+^ influx into neurons.

First, G-Rg1 can upregulate the anti-oxidant capacity of SOD, MPO, GSH-Px, and CAT, while simultaneously downregulating oxidative free radicals, such as ROS, RNS, and OH; it can also inhibit ischemic nerve damage and associated apoptotic effects (such as protein denaturation, enzyme inactivation, lipid membrane oxidation, mitochondrial oxidative respiratory chain damage, mitochondrial apoptosis induction, CC-3, Bal, and AIF) caused by oxidative stress and induced via the Akt, Nrf2/HO-1, PPARγ/HO-1, ERK, p38 and JNK MAPK pathways, the mitochondrial apoptosis pathway and the caspase-3/ROCK1/MLC pathway, thereby providing significant neuroprotective effects against cerebral ischemic injury.

Second, G-Rg1 can downregulate harmful inflammatory cytokines, such as IL-6, IL-1β, TNF-α, ICAM-1, and MMP-9, at both the protein and mRNA levels; upregulate anti-inflammatory factors regulated by NF-κB (p50 and p65) and IKK; inhibit the levels of PPARγ, Bax, CC-3, and CC-9 at both the protein and mRNA levels; and inhibit HMGB1 and the associated necrotic and apoptotic effects caused by oxidative stress (such as the activation of microglia and astrocytes in resident cells, the destruction of the blood-brain barrier caused by the inflammatory factors MMP-2, MMP-3, and MMP9, brain edema, loss of neuronal cells, and a large amount of ROS induced by excessive inflammatory responses) by regulating MAPK pathways, such as the JNK1/2, ERK1/2, and JAK1/STAT1 pathways, in addition to ERS, the HMGB1-induced TLR2/4/9 and RAGE pathways, and activate NF-κB, resulting in significant neuroprotective effects against cerebral ischemic injury. 

Third, G-Rg1 can increase the levels of cytokines that promote cell proliferation and differentiation, such as HIF-1α, EPO, VEGF, BDNF, and NGF, at both the protein and mRNA levels; promote angiogenesis and induce neurogenesis by regulating MAPK pathways, such as the JNK1/2 and ERK1/2, PI3K-Akt/mTOR, PKB/Akt, and HIF-1α/VEGF pathways; and affect ERS, resulting in significant neuroprotective effects against cerebral ischemic injury. However, the specific regulatory mechanisms that are affected in neurons and during angiogenesis remain unclear.

Finally, G-Rg1 can upregulate the energy metabolism capacity of Na-K-ATPase in addition to iNOS activity, ATP, AMP, total adenine nucleotides (TANs), and energy charge (EC); downregulate the free radical contents of Glu and asparaginic acid (Asp), modulated (inhibited) the influx of extracellular calcium and the release of intracellular calcium as well as nNOS activity; enhance the expression of GLUT3 and the activation of AMPKα1/2; and inhibit ischemic nerve damage and its associated apoptotic effects (such as intracellular calcium overload, AAA toxicity, energy metabolism disorder and mitochondrial apoptosis) via its effects on NMDA receptors, ERS, and the AMP/AMPK-GLUT pathways.

In summary, ginseng Rg1 is a tetracyclic triterpenoid derivative derived from natural medicinal plants that has significant and representative pharmacological activities. Additionally, in this overview, we show that GR promotes anti-ischemic stroke via its links to multiple pathways and its multitarget effects, as shown in Figure 2. On the one hand, its role and the results of relevant studies suggest potential strategies and novel methods that use multitarget and multilink combination therapy for the treatment of ischemic stroke; on the other hand, while these data provide a strong scientific basis for network pharmacology studies on natural medicinal plants, the pharmacological effects and mechanisms of active ingredients obtained from *Panax notoginseng* and ginseng have been comprehensively elaborated, a situation that is more conducive to the development and utilization of *Panax notoginseng* and ginseng. These findings provide ideas for research into the pharmacological actions and mechanisms of other active constituents, a reference for the rational clinical use of drugs, and scientific protection of resource utilization.

However, many of the actions and mechanisms of G-Rg1 remain unknown. These include its ability to regulate inflammation after the I/R activation of keratinocytes, and few studies have explored its effects on neurogenesis and autophagy regulation in brain nerve cells (Figure 2). At the same time, studies of the ginsenoside Rg1 have mostly focused on the effects of Rg1 on inhibiting apoptosis, while fewer cross-topic studies of multiple pathways have been performed. Therefore, it is worth exploring whether the ginsenoside Rg1 affects autophagy and inflammation-induced necrosis and whether its effects are protective or damaging to cerebral ischemia reperfusion so that we can obtain a more comprehensive understanding of the regulatory mechanisms used by G-Rg1 in the body.

## Figures and Tables

**Table 1:** Summary of G-Rg1-mediated mechanisms that regulate oxidative stress to exert neuroprotective effects on cerebral ischemia/reperfusion injury.

N	Model	Tissue Sites	Effects	Mechanisms	RF
1	Middle cerebral artery I/R injury model rats; OGD-induced cortical neuronal model	Cerebral I/R injury	↓ Indicators of oxidative stress and inflammation ↑ Neurological function; MPO and SOD activity, and CAT content ↓ Brain edema, TNF-α and IL-6 content	↑ Expression of PPARγ and NF-κB 65; Via PPARγ-regulated anti-oxidative and anti-inflammatory pathways	[24]
2	OGD-induced NSCs	Cerebral I/R injury	↓ OGD-induced apoptosis in NSCs ↓ OGD-induced oxidative stress ↓ Expression of CC-3 and Bax	↑ Expression of anti-apoptotic protein Bcl-2; ↓ OGD-induced oxidative stress; ↓ p38/JNK2 phosphorylation;	[36]
3	H_2_O_2_-induced SH-SY5Y cells	Ischemic cerebrovascular disease	↑ Survival rate of SH-SY5Y cells ↓ Amount of leaked LDH ↑ SOD activity	↓ Caspase-3 immunoreactivity; ↑ HSP 70 expression; Via anti-oxidation and inhibition of apoptosis;	[60]
4	OGD-induced BV2 microglial cells	Ischemic stroke	↓ NO level, TNF-α content and expression ↑ Cell viability, the content and expression of TGF-β1	Fcγ-receptor-mediated phagocytosis pathway;	[66]
5	H_2_O_2-_treated PC12 cells; TNF-α-treated EA.hy926 cells	Cardio-cerebral ischemic diseases	↓ H_2_O_2_-induced cytotoxicity ↑ Nerve cell survival rate	↓ Phosphorylation of NF-κB, p50, p65 and IKKα/β; Regulates the Nrf2/HO-1 pathway;	[61]
6	MCAO-induced SD rat models	Cerebral I/R injury	Effect similar to that produced by ROZ in activating PPARγ/HO-1	By activating PPARγ/HO-1 to protect against apoptosis and inflammation	[56]
7	BALB/c mouse t-MCAO model, H_2_O_2_-induced mouse cultured astrocytes	Cerebral I/R	↓ H2O2-induced apoptosis ↓ Ca ^2+^ overload, loss of MMP ↓ ROS production in astrocytes	Prevents astrocytes from undergoing apoptosis	[67]
8	MCAO/R-induced C57BL/6 mice	Cerebral I/R	↑ Nerve cell survival rate ↓ MDA and NO contents ↑ SOD activity and glutathione levels ↓ Nrf2 in the cytoplasm and ↑ in nucleus	↑ Nuclear translocation rate and HO-1 mRNA levels; By jointly activating the Nrf2/HO-1 signaling pathway	[62]
9	H_2_O_2_-induced PC12 cells	Oxidative stress-induced neuronal injury	↓ H_2_O_2_-induced cytotoxicity ↓ Phosphorylation and nuclear translocation of NF-κB/p65 ↓ Phosphorylation and degradation of IκB	↓ Activation of Akt and ERK1/2; Via anti-oxidative stress and the regulatory effects of H_2_O_2_ on the NF-κB pathway;	[59]

N: number, RF: reference, other abbreviations: as shown in the literature. (↓), downregulation or inhibition; (↑), upregulation or activation.

**Table 2:** Summary of the effect of G-Rg1 on the regulation of inflammation and its ability to exert neuroprotective effects on cerebral ischemia/reperfusion injury.

N	Model	Tissue Sites	Effects	Mechanisms	RF
1	Cerebral I/R-induced model, C57BL/6 mice	Cerebral I/R	↓ Infarct volume, neurological deficit scores ↓ IL-1β, TNF-α and IL-6 contents in serum ↓ Glu and Asp contents	↑ BDNF expression ↓ Expression of IL-1β, IL-6 and TNF-α in serum ↓ Glu and Asp contents	[1]
2	OGD-injured BV2 microglial cells, MCAO-induced male rat model	Focal cerebral ischemic stroke	↓ Neurobehavioral deficits, infarct volume, and brain edema ↓ CD11b-positive cell numbers and miR-155-5p levels	↓ Expression levels of miR-155-5p, pri-miR-155 and pre-miR-155 Inhibition of microglial miR-155-5p following ischemic injury	[2]
3	MCA I/R model in rats, OGD-induced cortical neurons	Cerebral I/R injury.	↓ Indicators of oxidative stress and inflammation ↑ Neurological function, CAT content, MPO, and SOD activity ↓ Brain edema, TNF-α and IL-6 contents	↑ Expression of PPAR-γ and NF-κB/65 PPARγ-regulated anti-oxidative and anti-inflammatory pathways	[3]
4	OGD-induced BV2 microglial cells	Ischemic stroke	↑ Expression levels of PPAR-γ, Bcl-2 ↓ Expression levels of CC-3, CC-9, IL-1β, TNF-α, HMGB1, and RAGE in the hippocampus	Fcγ receptor-mediated phagocytosis pathway	[6]
5	Cerebral I/R injury-induced C57BL/6 mouse model	Cerebral I/R injury	↑ Neurocyte survival rate ↓ Apoptotic rate, expression levels of CC-3 and ICAM-1, and TNF-α ↓ p-IκBα levels and nuclear translocation of NF-κB ↓ Phosphorylation of JAK1, expression of p-STAT1	↑ GRP78 expression ↓ Activation of NF-κB Anti-apoptotic and anti-inflammatory mechanisms JAK1/STAT1 signaling pathways and the regulation of ERS	[7]
6	H2O2-treated rat PC12 cells, TNF-α treated EA.hy926 cells,	Cardio-cerebral ischemic diseases	↓ NO level and protection of Bed cell viability ↑ Content and expression of TGF-β1 ↓ Content and expression of TNF-α	↓ Phosphorylation of NF-κB, p50, p65 and IKKα/β via the Nrf2/HO-1 pathway	[8]
7	MCAO-induced SD rat models	Cerebral I/R injury	Effect similar to that of ROZ in activating PPARγ/HO-1.	↓ Apoptosis and inflammation via the activation of PPARγ/HO-1	[9]
8	OGD-injured microglia model	mimics ischemia-injured microglia	↓ NO release ↑ TGF-β level ↓ TNF-α content	The up-regulation of TGF-β expression ↓ TNF-α expression	[10]
9	H_2_O_2_-induced PC12 cells	Oxidative stress-induced neuron injury	↓ Cytotoxicity induced by H_2_O_2_ ↓ Phosphorylation and nuclear translocation of NF-kB/p65 ↓ Phosphorylation and degradation of IκB	↓ Activation of Akt and ERK1/2 Anti-oxidative stress and the regulation of the NF-κB pathway	[11]

N, number; RF, reference; and other abbreviations are as shown in the literature. (↓), downregulation or inhibition; (↑), upregulation or activation.

**Table 3:** Summary of the effects of G-Rg1 on the regulation of the cell cycle, proliferation, differentiation, and regeneration in neurons, by which it exerts neuroprotective effects on cerebral ischemia/reperfusion injury.

N	Model	Tissue Sites	Effects	Mechanisms	RF
1	Modified Rice–Vannucci model	Hypoxia-ischemia brain injury	↓ Neurological impairment and pathologic damage ↑ Angiogenesis after HI	↓ CC-3 ↑ Expression of VEGF and HIF-1α signaling pathways	[12]
2	HIBD-induced SD rat model	Hypoxia ischemia brain damage (HIBD)	↓ Apoptotic index (AI) of neurons ↑ Protein expression of p-ERK1/2 and HIF-1α ↓ Expression of CC-3	↑ HIF-1α expression ↓ Activation of CC-3 by the Erk1/2 signaling pathway	[13]
3	I/R-induced SD rat model	Cerebral I/R injury	↓ Cell apoptosis ↑ ischemic conditions and cerebral recovery; ↓ Cell apoptosis and facilitating;	↓ Cell apoptosis ↑ Differentiation of BMSCs into neurons and glial cells	[14]
4	Cerebral I/R SD rat model	Focal cerebral I/R	↓ Changing trend in neurological deficit scores ↓ Evans blue content and aquaporin 4 expression	↓ Aquaporin 4 expression	[14]
5	MCAO and 24-h reperfusion SD rat model	Cerebral I/R	↓ Scores on neurofunction and the apoptosis rate ↑ Number of surviving pyramidal cells ↓ Expression of p-JNK	↑ Expression level of p-ERK1/2 ↓ Neuronal apoptosis Regulation of the expression levels of p-ERK1/2 and p-JNK	[16]
6	HIBD-induced SD rat model	Hypoxia-ischemia brain damage	↑ Protein expression of HIF-1α and VEGF ↑ Number of vwf-positive cells	↑ Angiogenesis after HIBD ↑ Strengthening and stabilizing the HIF-1alpha/VEGF signaling pathways	[17]
7	HIBD-induced SD rat model	HIBD	↑ Neural viability ↑ Angiogenesis ↑ Neurogenesis	↑ Potential regulator of HIF-1α expression	[18]
8	Normal adult mice, global ischemia gerbil model	Cerebral I/R	↑ Proliferation and differentiation of neural progenitor cells ↑ Neural plasticity in efficacy and structure	↑ Expression of BDNF, Bcl-2 and anti-oxidant enzymes ↑ New synapse formation ↓ Apoptosis and calcium overload	[19]
9	Male Mongolian gerbils	Transient global ischemia	↑ Proliferation of cells ↑ Number of surviving BrdU-positive cells	↑ Proliferation of cells in the SGZ of adult gerbils at 11 DAI	[20]

N, number; RF, reference; and other abbreviations are as shown the literature. (↓), downregulation or inhibition; (↑), upregulation or activation.

**Table 4:** Summary of the ability of G-Rg1 to regulate energy metabolism, endoplasmic reticulum stress, neurotransmitters, and blood-brain barrier permeability, whereby it exerts neuroprotective effects on cerebral ischemia/reperfusion injury.

N	Model	Tissue Sites	Effects	Mechanisms	RF
1	CI/RI-induced- C57BL/6 mouse model	Cerebral ischemia-reperfusion injury (CI/RI)	↑ ATP, ADP, and AMP contents and the level of TAN ↑ Phosphorylation of p-AMPKα1/2	↑ mRNA and protein levels of GLUT3 ↑ Uptake of glucose into nerve cells via AMPKα1/2	[96]
2	CI/RI-induced-C57BL/6 mouse model	Cerebral ischemia-reperfusion	↑ Neurocyte survival rate ↓ Apoptotic rate and CC-3 levels ↓ Level of TNF-α and ICAM-1 mRNA ↓ Phosphorylation of IκBα and JAK1 and expression of p-STAT1 ↓ Rate of nuclear translocation of NF-κB	↓ Activation of NF-κB and JAK1/STAT1 pathways ↑ GRP78 expression via anti-apoptosis and anti-inflammation The regulation of ERS after cerebral ischemia	[75]
3	OGD-induced BV2 microglial cells and N2a neuronal cells,	Cerebral ischemia-reperfusion	↓ LDH leakage ↑ Neuronal cell viability ↑ Mitochondrial ultrastructure	↓ Expression of NMDA receptor subunit 1 and activated caspase-3	[97]
4	Meta-analysis, animal models of focal cerebral ischemia	Human ischemic stroke	↓ Infarct volume ↑ Neurological function scores	G-Rg1 exhibited marked efficacy against acute ischemic stroke	[39]
5	MCAO model	Focal cerebral ischemia/reperfusion	↓ Neurobehavioral function scores and infarct volume ↓ Permeability of the BBB	↓ Expression of PAR-1	[38]
6	OGD/R-induced cultured hippocampal neurons	Cultured hippocampal cells	↓ Cell viability loss and cell apoptosis ↓ nNOS activity and free Ca^2+^ concentration	↓ Calcium over-influx into neuronal cells ↓ nNOS activity	[98]
7	Transient global ischemia-induced adult gerbil model	Transient global ischemia	↑ iNOS activity ↑ Hippocampal progenitor cell proliferation	Activation of iNOS activity and NMDA receptors	[99]

N, number; RF, reference; other abbreviations as shown in the literature. (↓), downregulation or inhibition; (↑), upregulation or activation.
